# Pathogenic Microorganisms Associated With Childhood Diarrhea in Low-and-Middle Income Countries: Case Study of Yaoundé – Cameroon

**DOI:** 10.3390/ijerph5040213

**Published:** 2008-12-02

**Authors:** HB Nguendo Yongsi

**Affiliations:** Department of Nursing and Human Sciences, University of Chicoutimi/555, boulevard de l'Université Chicoutimi (QC), G7H 2B1, Canada; E-Mail: Blaise_nguendo-yongsi@uqac.ca; Tel.: +1-905-817-1523

**Keywords:** Diarrhea, pathogens, microorganisms, children, Yaoundé, Cameroon

## Abstract

Notwithstanding significant advancement in the understanding of pathogenesis and management, diarrheal illnesses remain one of the principal causes of global childhood mortality and morbidity. Infections account for most illnesses, with pathogens employing ingenious mechanisms to establish disease. In 2002, an interdisciplinary program “Populations *et al.* Espaces à Risques SANitaires” (PERSAN) was set up under the patronage of the Development Research Institute (IRD). Focused on health in Cameroon’s urban environment, the program mainly sought to identify diarrhea risk factors in Yaoundé. So for, a cross-sectional epidemiological study in children aged 6–59 months was carried out using a standardized protocol. The survey was initiated in 2002 and conducted during April to June in the year 2005. 3,034 stool samples were collected from children in twenty neighbourhoods in Yaoundé and examined at the Epidemiology and Public Health Laboratory of the Cameroon Pasteur Institute. About 60% of the patients were aged less than two years and 52% were male. Among the 437 patients with the diarrheal disease, 260 were found to be of infectious etiology, i.e. micro organism was detected in 59.5 % of the cases. Out of which, 10 (03.8%), 96 (36.9%), and 154 (59.2%) were respectively caused by pathogenic viruses, pathogenic bacteria and pathogenic parasites. Higher prevalence was found in overcrowded and under supply spontaneous settlement (78.4%) than in less crowded and formal residential settlement (21.5%). Etiologic data on diarrheal diseases and their spatial distribution are important tools for public health management and control strategic planning.

## 1. Introduction

Diarrheal diseases have been described as far back as the ancient Greek civilization [1, 2]. For instance, Hippocrates’ writings (460–370 B.C.) were likely referring these diseases, describing them as “*abundant liquid stool at short intervals*” [3, 4]. During the Middle Ages, terms including *Rheuma Gastros* (running stomach) and *Rheumatismus* (stomach ache) used respectively by Gallien [5] and Alexandre de Tralles [6], focused on the physiopathological mechanism of the disease. In year 30, Celse revealed more clinical elements of the disease: «. . . *the patient excretes blood which is usually mixed up with liquid excreta and at other times with mucus*» and considered diarrhea as «*an illness in which intestines are not to able to retain anything, and in which almost everything in the alimentary canal is lost as soon as it is eaten without digestion*» [7]. Following Celse’s contribution, Gallien established inappropriate hygiene as the cause of diarrhea in his theory of “*aqueous humour*”, stating that toxic substances were present in the stomach and that diarrhea was a mechanism of evacuation. This remained as the primary etiologic theory for centuries. Towards the middle of the 17th century, Fernel classified diarrhea as an intestinal disease in the same category with intestinal blockages, gastric ulcers, and amebiasis. Like Gallien and Fernel, Grall *et al*. also subscribed to the view suggesting that poor food hygiene was the cause of diarrhea [8]. The first descriptions of diarrhea in developing countries date back to 1883 when *Vibrio cholerae* was isolated in Egypt during the third 1865 world cholera epidemic [9, 10]. It is this third great epidemic that left endemic hubs; some of which have endured to date. Unfortunately, a lack of information prevents an appreciation of the disease evolution since the discovery of the vibrion in Egypt until the middle of the 20th century. The first accurate data on the epidemiology of diarrhea date back to the 1960s. According to the WHO, a specialized team worked in seven developing countries from 1960 to 1965 and reported that the monthly prevalence rate among children less than 6 years of age was 40% [11]. Studies carried out in diverse regions of the world in order to appreciate accurately the magnitude and gravity of the problem led to the estimation that 500 million children had undergone one or more diarrhea episodes, and 4.6 million deaths had occurred from it in 1980 [12]. Thirty years later, estimates still report a high incidence and prevalence of diarrhea.

According to the World Health Organization, each year there are an estimated 4 billion cases of diarrhea. In 2007, 1.8 million people died worldwide from diarrheal diseases (including cholera), with 88% of these diarrheas being attributable to unsafe water supply, inadequate sanitation and hygiene. They were the second leading cause of disability-adjusted life years (DALY) lost and were the third leading cause of death (4% of all deaths) [13]. Presently, the risk of contracting diarrheal diseases are increased 5-fold in sub-Saharan Africa compared to developed countries (39.1% vs. 7.2%, respectively) [14]. Thus, diarrheas are an imminent threat to public health, particularly in the developing countries where it is one of the main causes of morbidity and mortality in children under 5 years of age (90%) [15]. In those countries, the average number of episodes of diarrhea per child per year within this age group is 3.2, and 21% of childhood mortality in children is associated with diarrhea, resulting in nearly two million deaths per year. In sub-Saharan Africa, mortality caused by acute diarrhea varies from 1.9% of all deaths in Gambia to 37% in Nigeria, with most of the deaths occurring during the first year of life [16–18]. Apart from well-described enteropathogens such *Shigella* spp., *Salmonella* spp., and enterotoxigenic such *Esherichia coli*, there are also a wide variety of viruses and protozoa that cause diarrhea in those endemic areas. Although diarrhea are recognized as a serious public health problem in Cameroon, no comprehensive studies relating diarrhea-causing pathogens responsible for endemic diarrhea among Cameroon’s children have been reported in the medical literature. The aim of this study is to describe the main etiologic infectious agents of diarrhea in Yaoundé and their distribution within the urban space.

## 2. Material and Methods

### 2.1. Location of study

**Figure 1:**
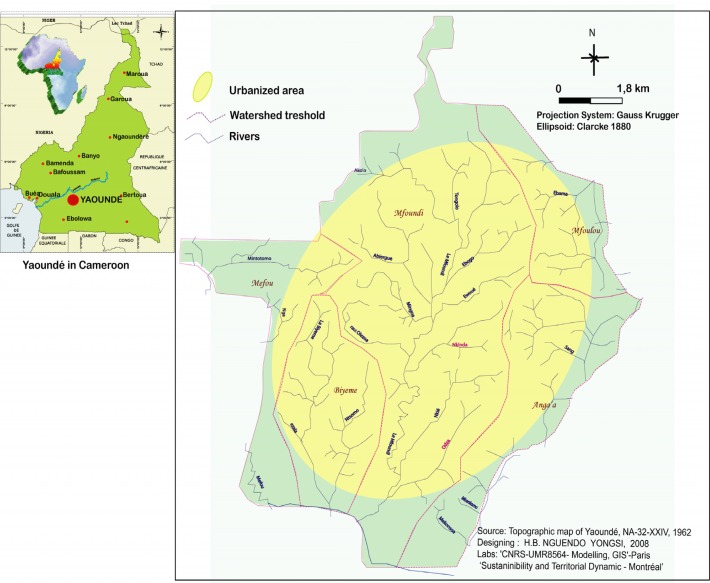
Location of Yaoundé, Cameroon.

This study took place in Yaoundé, the capital of Cameroon, situated in Central Africa between latitudes 3° 47′ and 3° 56′ North and 11° 10′ and 11° 45′ East (Figure 1). Yaoundé displays the classical Equatorial Guinea climate (regular and abundant rainfall of more than 1,600 mm per annum, and a fairly high average annual temperature of 23°C). Like many sub Saharan African cities, Yaoundé is currently experiencing very rapid urbanization. In 1926, date of the first population census, Yaoundé had 100,000 inhabitants. With an estimated annual growth rate of 4.5 percent since 1980, urban population has grown from 812,000 inhabitants in 1987 to 1,500,000 inhabitants in 2000, and to about 2,100,000 in 2006 [19]. However, this population growth has not been monitored by the city planners and decision makers. Consequently local authorities have failed to provide neighbourhoods with adequate utilities, services and infrastructure. Therefore, city dwellers are facing difficulties such as getting access to improved water sources, to basic sanitation, and to a relatively good personal and domestic hygiene [20].

### 2.2. Data collection

*Definition of diarrhea and nutritional status assessment*: Diarrhea is generally characterized by the frequent occurrence of watery stools. In a technical sense, however, it is more difficult to define because the suggested elements which are asymptomatic of the definition of diarrhea vary according to the objectives of each study [21–24]. Following clinical signs, we have considered diarrhea as the sudden and frequent occurrence of abundant and consistently abnormal watery or mucus stools more than three times a day and more than 300 g per stool. The stools should be mixed with a phlegm-like substance or blood, and are associated with dysentery. To indicate its acute character, the episode must last for about 14 days. As far as nutritional status is concerned, it was assessed by weight for age Z-score. Severe malnutrition was defined as a weight for age Z-score < - 3, moderate malnutrition was between -3 and -2, and mild malnutrition between -2 and -1. Well-nourished children were considered when at < -1 Z-score.

*Patients:* To minimize the risk of confusion between the infectious diarrhea and the soft feces normally observed in babies, the survey targeted only children from 6 to 59 months. Thus, the childless household and those with children outside the age group were eliminated from the sample. In the households with several children in the survey age bracket, a table of random numbers was used to select only one of them.

**Figure 2:**
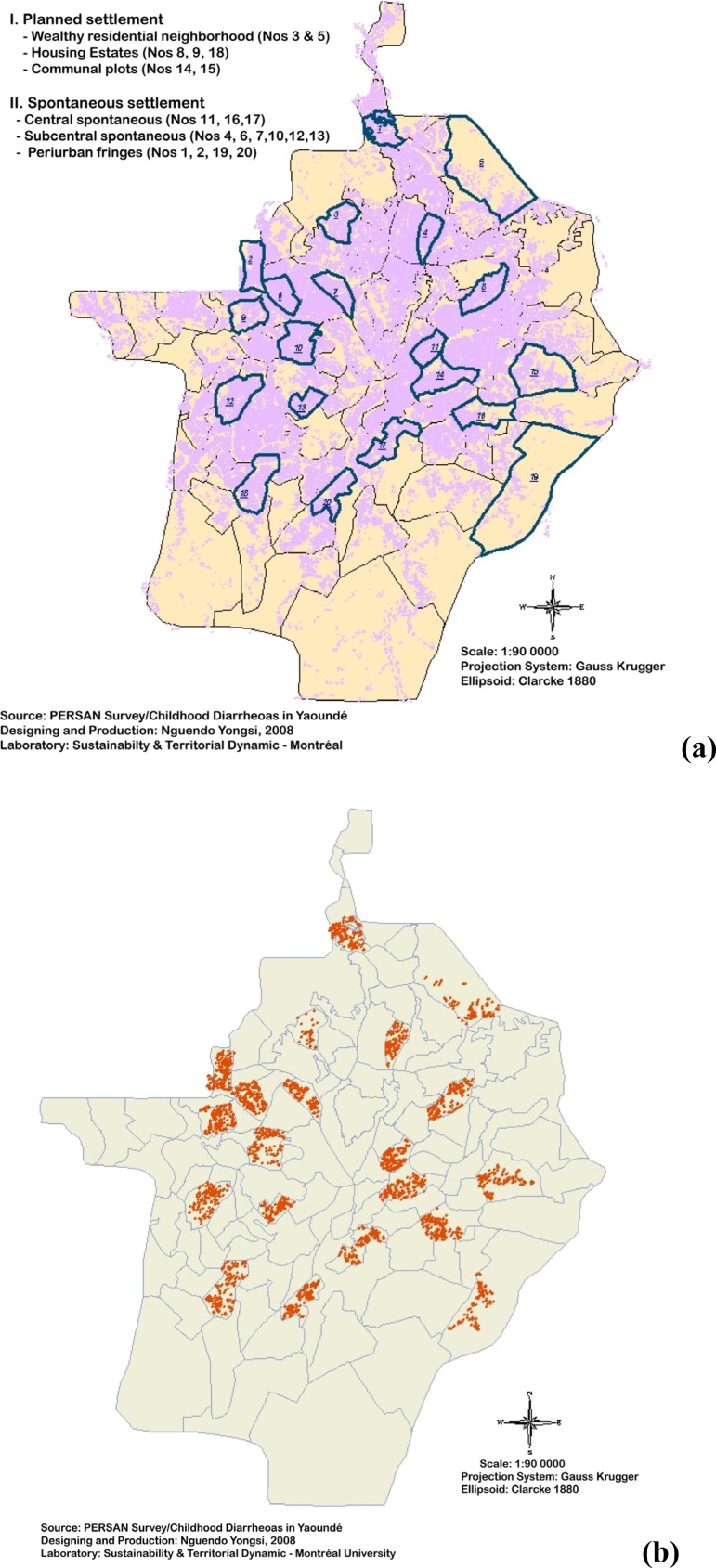
**(a)** Selected neighbourhoods. **(b)** Surveyed households with < 5 years Children.

*Field survey:* The area has two wet seasons (April to June and August to October). Because during the rainy season, many microorganisms are more prevalent and the cases of diarrhea increase, the study was conducted during the first rainy season i.e. April to June 2005. The survey used a stratified random sampling procedure based on two stages to select targeted neighborhoods. First, 20 neighborhoods were selected out of the 105 that make up the city (Figure 2a). They were representative of the six types that the city contains. Second, 3,034 households qualified for the final survey was selected (Figure 2b). In the selected neighborhoods and households, we used the quantitative method through a structured questionnaire. Under our coordination, the questionnaire was administered by a team of final year medical doctors from the Faculty of Medicine and biomedical sciences of the University of Yaoundé I. Approved by the Cameroon National Ethics Committee, this medical survey aimed (i) to examine children’s clinical history, physical signs, and previous treatments, (ii) and to detect cases of diarrhea among them. Thus, when a case of diarrhea was reported, a physician visited the home, interviewed the mother or caretaker of the child, examined the child and collected a fresh stool. The stool specimens were placed on wet ice and immediately taken to the Cameroon Pasteur Institute where they were dispatched to the bacteriological, virological and parasitological laboratories for etiological studies. Each positive sample was correlated with the neighbourhood pertaining to the household to which the infected child belonged to. According to current WHO regulations, diarrheic children were treated with oral rehydration therapy and with appropriate antihelminthics and antibiotics selected on the basis of the culture results.

*Processing of samples: (1) Bacteriology:* After reception, stool samples were cultured in various solid selective media and selenite broth. Plates were incubated for 18 hours at 37°C, except for *Campylobacter* medium which was incubated 48 hours at 42°C under microaerophilic conditions. After 24 hours of incubation, the selenite broth was subcultured onto *Salmonella-Shigella* agar. Colonies were identified based on morphologic characteristics. When suspected to be relevant, identification was done using the appropriate tests, followed by an API20E powered by BioMerieux laboratory. *Salmonella, Shigella,* and *Vibrio* strains were agglutinated using specific antiserum. Identification of the different diarrhagenic *Escherichia coli* (EPEC, ETEC, EAEC, VTEC) was done by standard methods formerly made known by Vargas *et al.*[25] and recently described by Moyo *et al.*[26] and Mandomando *et al*[27]. *Campylobacter* spp. were identified based on morphologic characteristics and on oxidase test, and confirmed by Gram staining. *(2) Virology:* A sample of each stool was frozen in a saline solution and tested by enzyme-linked immunosorbent assay for rotavirus antigen as presented in the Meridian Bioscience kit [28] and well described by Dennehy *et al.*[29]. The reagens were supplied by the Sanofi Diagnostic Pasteur collaborating center in Paris. Enteric adenovirus were investigated using SDS/EDTA-pretreated filter paper strips well described by Zlateva *et al.*[30]. *(3) Parasitology:* Each fresh stool was examined microscopically for *leucocytes*, trophozoites of *Entamoeba histolytica*, and *Giardia lamblia* by using a saline and iodine preparation [31]. Kato and migration inhibition factor techniques were used for determination of concentrations. *Cryptosporidium parvum* and *Cyclospora cayetanensis* were detected with a modified acid-fast stain [32, 33]. Campylobacter strains were isolated and identified by standard tests and the PCR method [34]. Children with fever or having an episode of fever within the previous 24 h were tested for malaria parasites in blood.

*Data management and statistical methods:* All data were entered into Access database and locked after checks for internal and external consistency. Values of variables were counted and summarized in tables of frequency. The *x*^2^ test was used for the analysis of categorical variables. A *P* value of ≤ 0.005 was considered significant.

## 3. Results

*Epidemiologic features:* A total of 3,034 children aged up to five years were enrolled in the study. Among these, diarrhea was one of the diagnoses in 437 (14.4%) (Table 1).

*Samples* were collected from all of them (100%). Seventy two (16.47%) were younger than 12 months of age, and two hundred ninety eight (68.19%) were between 12 and 36 months of age (Table 2).

**Figure 3:**
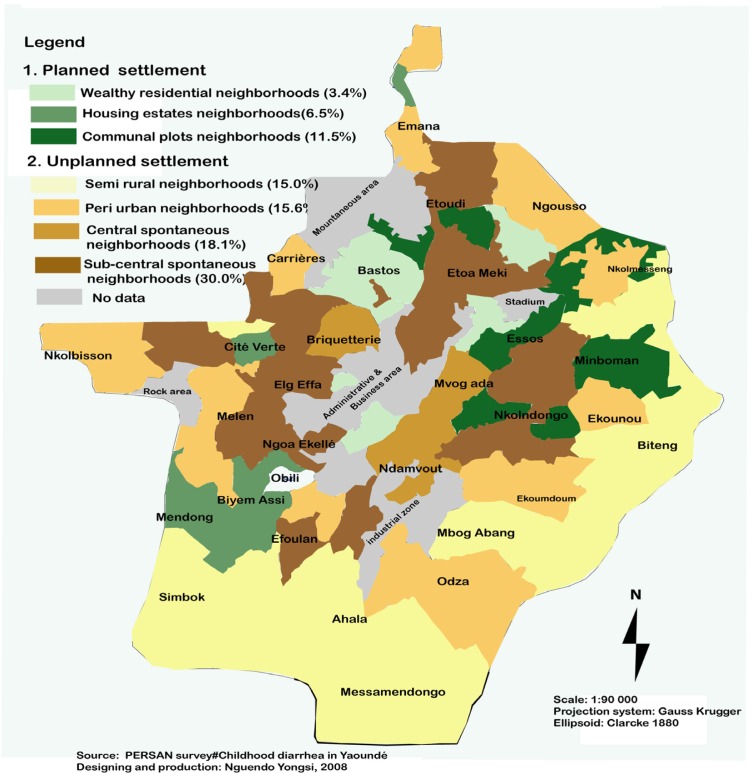
Spatial distribution of diarrheas in Yaoundé, according to type of neighbourhoods.

*Co*-infections were found in one hundred and twenty five children, accounting for 48.1% of the total number of positive cultures. Of these positive samples, four (3.2%) were virus/parasite co-infections, one hundred and five (84%) were bacteria/parasite, and sixteen (12.8%) were virus/bacteria/parasite co-infections. Three hundred fifty eight (81.9%) children were males, i.e. a male-to-female ratio of 1:4.5. One hundred ninety nine parents (45.5%) were thinking of seeking medical attention (hospital) for their sick children, whereas ninety five (21.7%) were thinking of resorting to street pharmacists (street medicines dealers), eighty six (19.6%) to traditional remedies, and fifty seven (13.1%) did not have any therapeutic choice. Low level of diarrhea (Fifty six, 21.5%) was identified in formal i.e. planned settlement, whereas high level of diarrhea (two hundreds and four, 78.4%) was found in children living in unplanned settlement, namely in central and sub-central spontaneous neighbourhoods (Figure 3).

*Etiologic agents:* On the 437 samples examined, a potential infectious pathogen was isolated from 260 (59.5%). Pathogenic viruses were isolated from 10 (3.8%) samples, bacteria from 96 (36.9%), and parasites from 106 (59.2%) (Table 3).

Those pathogens were unequally found among the children. *Enteric adenovirus* (02.7%), *Salmonella* spp. (09.6), *Campylobacter* spp. (08.8%), *Esherichia coli* - enteropathogenic (04.2%), *Shigella spp.* (07.3%), *Ascaris lumbricoides* (17.8%), *Giardia lamblia* (13.2%), *Trichuris Trichiura* (10.7%), and *Entamoeba* (08.4%) were the most frequently found pathogens (Table 4). *Rotavirus* was found in only three children, all younger than 23 months. The presence of *Salmonella* spp. (P < 0.005) or *Campylobacter Jejuni* (P < 0.003) was statistically related to age, being more frequent among children between 12 and 35 months of age; whereas *Ascaris Lumbricoides* (P < 0.001) and *Trichuris trichiura* (P < 0.003) were more frequent in children between 12–59 months of age. Though not significant, *Shistosoma intercalatum* was the very rare pathogen to be isolated.

*Clinical features*: In all children having had a microorganism isolated or not, fever and cough were the most recurrent clinical finding, whereas vomit and pains were frequently found only among children with *Ascaris lumbricoides, Giardia lamblia, and E. coli (EPEC, EAEC).* Persistent diarrhea i.e. diarrhea that continued for longer than 14 days occurred in 14.41% (63 of 437). These sixty three children were between 6 and 23 months of age and were infected with two or three pathogens. Analysis of fecal samples in children with diarrhea showed watery diarrhea in 72.3%, diarrhea with mucus in 20%, diarrhea with blood in 3.1% and diarrhea with mucus and blood in 1.6 % of all patients. From the two hundred and sixty children with infectious diarrhea, two hundred and two children (77.7%) were diagnosed with malaria. Only ninety seven (22.2%) children were well-nourished. Whereas sixty nine (15.8%) had severe malnutrition, one hundred and one (43.7%) had moderate malnutrition, and eighty (18.3%) had mild malnutrition. Other Clinical features included symptoms of upper respiratory tract infection (21.9%), and abdominal pain (12.5%).

## 4. Discussion

This study, which covered a 3-months period, was the first direct field investigation in Cameroon of pathogenic agents associated with diarrhea in children. The results found in the present study are in whole similar to those reported from other countries where diarrheal diseases are recognized as a major cause of morbidity and mortality in children under five years of age [35]. Our male-to-female ratio of 1:4.5 is close to that of several studies which reported higher number of male patients as compared to female patients to have suffered from diarrhea [36–38]. In this study, at least one pathogenic microorganism was isolated from 59.5% (260/437) of the stools of children with diarrhea. In spite of Mandomando and Sur [23–39] who reported a low rate (42.2%), this percentage is similar to previous studies carried out and which well correspond with health realities in developing countries [40–42].

It is difficult to draw conclusions about rotavirus because of the low rate of detection of this virus. However, this result is not surprising because usually during the rainy season, percentages of diarrhea due to rotavirus are lower than during the dry season [43, 44]. On the other hand, rotavirus has been associated with approximately one quarter of all diarrhea in South Africa [45]. Group A rotavirus is the most common human strain, with a worldwide distribution, whereas Groups B and C are mainly found in East and South-East Asia [46, 47]. No data on the prevalence of the different groups are not available for Cameroon. An explanation would be that cases of rotavirus either were not severe to cause diarrhea or had short duration. Thus, there’s a need for longer studies with adequate methodology in order to determine the real rotavirus prevalence in Cameroon. However, Enteric adenovirus has displayed a high prevalence compared with rotavirus. This can be due to their epidemiologic feature: notwithstanding the types, all are transmitted by direct contact, fecal-oral transmission, and occasionally waterborne transmission. Some types are capable of establishing persistent asymptomatic infections in intestines of infected hosts and infections can occur throughout the year [48]. Case of Enteric adenoviruses serotypes 40 and 41 that we isolated, mainly in stools of children between 5 and 12 months of age (57.14%).

Despite Gascon and Lindblom [49, 50] who described a low percentage of *Campylobacter jejuni* isolated in children, our results show a different pattern - probably because our study design has included children from one to five years, an age strata at which *Campylobacter* is more prevalent. The relatively lower frequency of *Salmonella* spp. found in our study is consistent with that of African studies in which they were isolated in less than 10% of children younger than five years of age with diarrhea [51, 52]. These salmonella are likely to adapt to domesticated cattle and all varieties of fowls and chickens. The reservoir of infection in animals constitutes the principal source of diarrhea caused by *Salmonella* spp*. Shigella* spp. has also been enteropathogens statistically related with an increased risk of diarrhea in under five years children in Yaoundé. Asymptomatic infection was common and may have arisen due to prolonged excretion of organisms [53]. Its relatively low prevalence in our study may be explained by the fact that it included severe cases requiring hospitalization. Yet, we did not investigate hospitalized children. Furthermore, *Shigella* spp. usually appears in outbreaks, so that in the absence of an epidemic, it is not frequently isolated [54].

As well as other enteropathogens, *Diarrheagenic E. coli* were frequently isolated, supporting the well-documented role of this sp. in diarrheal disease [55, 56]. The main *E. coli* pathotype found was *Enteropathogenic Escherichia coli* (EPEC, 04.2%). The picture is similar to a study in Argentina, where EPEC was isolated from nine cases (04.5%) of a total of 200 diarrhea patients [57]. EPEC is a well known enteropathogen. Together with *Enterotoxigenic Escherichia coli* (ETEC), they cause an important number of cases of diarrheal illnesses with dehydration [58]. However, in some studies, ETEC and EPEC strains were not related to diarrheal illnesses [59, 60] As far as *Enteroaggregative Escherichia coli* (EAEC) strains, they were first described as a putative virulence group by Nataro *et al*. [61]. In our study as well as in others [62, 63], EAEC was not related to diarrhea. This might be so because hosts factors like vertically acquired antibodies or breast milk could have played a role in the pathogenesis of diarrhea whether acute or persistent [64]. Another hypothesis might be an unexpectedly long period of EAEC strain excretion in feces after a diarrhea episode treated with only rehydration salt and exceeding the two weeks without diarrhea required for controls [65]. The low frequency of verotoxin-producing *E. coli* (VTEC, 01.5%) reported in our study is consistent with others studies in low and middle income countries [66]. In those countries, prevalence of VTEC in childhood diarrhea is lower than that of ETEC, EPEC, or EAEC.

Intestinal nematodes seem to play a great role in diarrheal diseases in this area. The high rate of parasites isolated from stools samples suggests a high prevalence and even incidence of these health problems in the Yaoundé urban and rural region for children at this age. The epidemiologic data suggest that there is an age dependency in the isolation or parasites, especially in the case of *Ascaris lumbricoides* and *Trichuris trichiura*, both being more frequently isolated in children older than twelve months, and statistically related to diarrhea. This could be because, children older than twelve months of age are in permanent contact with soil and thus more prone to infection, and have non hygienic feeding habits. In short and as reported in Venezuelan and Brazilian children [67, 68], the high and statistically significant frequency of enteric parasites in our study is conclusive of their role in diarrheal diseases. Also, the fact that *Entamoeba histolytica*, *Giradia lamblia*, and *Trichomonas intestinalis* are a cause of diarrhea here is a well-documented fact [69].

Another observation from the present study is that infection with multiple pathogens was very common. This is the reflection of environmental contamination [70–72], but it makes it difficult to identify which pathogen is the real causative agent of an episode of diarrhea. It might also be that multiple microorganisms act synergistically to produce diarrhea. The presence of fever in most diarrheic children could be caused by malaria, as the disease is endemic in the area and that nearly 77.70% of the children were positive with *Plasmodium falciparum* parasites. Vomiting was frequent in children with *Giardia lamblia*, *Ascaris lumbricoides*, *Enteric Adenovirus*, and *E. coli*. Blood noticed in the stools might be caused by *Shigella* spp. as its capacity to penetrate and multiply within the intestinal mucosa, causing ulceration is well known [73].

Despite the recognition of severe ill effects of the disease and mostly the enormous amount of resource being spent to control their spread [74], diarrhea still remains a serious threaten in many urban communities for a number of reasons: high incidence of malnutrition and infections, overcrowding of children in one house, lack of personal hygiene, lack of piped water and sanitation, widespread faecal contamination of the environment, and poor quality of housing [75–77]. In Yaoundé, this is particular obvious in informal settlement namely in central/sub-central and Peri urban neighbourhoods’ with poorest living conditions and consequently with a higher level of diarrhea (≥ 78%). However, formal settlements such as wealthy residential neighbourhoods and housing estates provided with all the urban utilities and infrastructure [78] display a lower level of diarrheas. This finding that levels of diarrhea diseases vary from one neighbourhood to another within the urban space might be a reflection of changing patterns of urban settlement.

## 5. Conclusion

Diarrheal diseases are one of the main health problems in Cameroon, as in many low-and-middle income countries. Clarification of the microorganisms associated with diarrhea in Cameroon is an essential step toward the implementation of effective primary health care activities against the disease. The present etiological study is also meaningful in that it provides information on the prevalence of enteropathogens in various neighbourhoods. Thus, this study opens door to further investigations aiming at analyzing specific epidemiology and virulence of diarrhea-causing pathogens in Cameroon. Therefore, suitable and efficient local control strategies can be set up. From a methodological point of view, analysis of diarrhea-causing pathogens according to neighbourhoods is a proof that a spatial epidemiological study at urban districts scale can be relevant in developing urban settings as whole. Such study is urgently required to disaggregate urban health information into smaller and manageable districts so as to guide local decision making on urban health. The contextual level of analysis allows showing that levels of diarrhea differ across the city. For example, Figure 3 shows that diarrheal prevalence varies across the city neighbourhoods. From these intra-urban variations, the learning objective is that, contrary to the existing literature on African urban environments, all the poor are seldom at the same risks in an urban neighbourhood. In health planning, such knowledge can be useful for setting suitable and effective strategies disease and health intervention strategies that are able to adequately address neighbourhood health determinants.

## Figures and Tables

**Table 1:** Distribution of diarrheal diseases, according to gender in Yaoundé.

Gender	Surveyed individuals	Cases of diarrheas
Male	1,564 (51.6%)	358 (81.9%)
Female	1,470 (48.4%)	79 (18.1%)
Variable coverage	3,034 (100.0%)	437 (100.0%)

Source: PERSAN Survey/Infant diarrhea in Yaoundé, 2002 & 2005

**Table 2:** Age strata of 437 children with diarrhea in Yaoundé.

	Diarrheal disease?	
Strata	Yes	No
06 - 11 months	72	351
12 - 23 months	178	628
24 - 35 months	120	709
36 - 47 months	43	530
48 - 59 months	24	379
Total	437	2,597

Source: PERSAN Survey/Infant diarrhea in Yaoundé, 2002 & 2005

**Table 3:** Infectious agents identified in diarrheic faeces.

Causative agents	Genus	Frequency	Percentage
Viruses	*Rotavirus*	3	01.1
	*Enteric Adenovirus*	7	02.7

Bacteria	*Salmonella* spp.	29	11.2
	*Shigella* spp.	23	08.8
	*Escherichia coli*	19	07.3
	*Campylobacter jejuni*	25	09.6

Parasites	*Ascaris lumbricoides*	46	17.8
	*Trichuris trichiura*	28	10.7
	*Entamaeba*	22	08.4
	*Giardia intestinalis*	34	13,2
	*Trichomonas*	8	03.2
	*Shistosoma*	5	01.9
	*Chilomastix*	4	01.5
	*Cryptosporidium*	7	02.6

Total		260	100.0

Source: PERSAN Survey/Infant diarrhea in Yaoundé, 2002 & 2005

**Table 4:** Frequency and age distribution of micro organisms isolated from fecal samples of children with diarrhea.

Microorganisms	Prevalence		06– 11 months		12–35 months		36–59 months		*P* value
	*N*	%	*N*	%	*N*	%	*N*	%	
*Rotavirus*	3	01.1	1	33.3	2	67.6	0	00.0	NS
*Enteric adenovirus*	7	02.6	4	57.1	3	42.8	0	00.0	<0.008
*Salmonella* spp.	25	09.6	6	24.0	14	56.0	5	20.0	<0.005
*Shigella* spp.	19	07.3	4	21.0	9	47.3	6	31.5	<0.004
*Campylobacter* spp.	23	08.8	3	13.0	12	52.1	8	34.7	<0.003
*E. coli-EAEC*	8	03.1	5	62.5	3	37.5	0	00.0	NS
*E. coli-ETEC*	6	02.3	3	50.0	2	33.3	1	16.6	NS
*E. coli –EPEC*	11	04.2	2	18.1	8	72.7	1	09.0	<0.001
*E.coli-VTEC*	4	01.5	0	00.0	2	50.0	2	50.0	NS
*Ascaris lumbricoides*	46	17.8	3	06.5	15	32.6	28	60.8	<0.001
*Trichuris trichiura*	28	10.7	0	00.0	19	67.8	9	32.1	<0.003
*Entamoeba histolytica*	22	08.4	3	13.6	9	40.9	5	22.7	NS
*Giradia lamblia*	34	13.1	11	32.3	14	41.1	9	26.4	<0.002
*Trichomnas intestinalis*	8	03.1	0	00.0	3	37.5	5	62.5	NS
*Shistosoma*	1	00.3	0	00.0	0	00.0	1	100	NS
*Shistosoma mansoni*	4	01.5	0	00.0	4	100	0	00.0	NS
*Chilomastix mesnili*	4	01.5	0	00.0	3	75.0	1	25.0	NS
*Cryptosporidium*	7	02.6	2	28.5	4	57.1	1	14.2	<0.008
